# Local Treatment of Acute Throat Affections

**Published:** 1908-05-16

**Authors:** J. W. Miller

**Affiliations:** 125 Whalley New Road, Blackburn


### Editor's Letter-Box.

**Our correspondents are reminded that prolixity is a great bar to publication, and that brevity of style and conciseness of statement greatly facilitate early insertion.**

### LOCAL TREATMENT OF ACUTE THROAT AFFECTIONS.

Sir,—In my article on "Local Treatment of the Commoner Acute Throat Affections" in The Hospital of to-day's issue, menthol gr. $\frac{1}{12}$ should have appeared along with the other substances in the formula referred to, which can be obtained in tablet form; one tablet to be crushed and mixed with four ounces of warm water before use.

Yours faithfully,

J. W. Miller.

125 Whalley New Road, Blackburn, May 9, 1908.

